# Genomic modifications for enhanced antibiotic production in rifamycin derivative-producing *Amycolatopsis mediterranei* S699 strains: focusing on *rifQ* and *rifO* genes

**DOI:** 10.3389/frabi.2024.1399139

**Published:** 2024-06-24

**Authors:** Moritz Müller, Elena Bialas, Irina Sturm, Utkarsh Sood, Rup Lal, Andreas Bechthold

**Affiliations:** ^1^ Institute of Pharmaceutical Biology and Biotechnology, Albert-Ludwigs-Universität, Freiburg, Germany; ^2^ Department of Zoology, Kirori Mal College, University of Delhi, Delhi, India; ^3^ Acharya Narendra Dev College, University of Delhi, New Delhi, India

**Keywords:** rifamycin, *Amycolatopsis mediterranei* S699, tuberculosis, increased antibiotic production, homologous recombination, *rifQ*, RifO

## Abstract

Rifamycin and its derivatives are natural products that belong to the class of antibiotic-active polyketides and have significant therapeutic relevance within the therapy scheme of tuberculosis, a worldwide infectious disease caused by *Mycobacterium tuberculosis*. Improving the oral bioavailability of rifamycin B was achieved through semisynthetic modifications, leading to clinically effective derivatives such as rifampicin. Genetic manipulation of the rifamycin polyketide synthase gene cluster responsible for the production of rifamycin B in the *Amycolatopsis mediterranei* strain S699 represents a promising tool to generate new rifamycins. These new rifamycins have the potential to be further derivatized into new, ideally more effective, clinically usable compounds. However, the resulting genetically engineered strains only produce these new derivatives in low yields. One example is the strain DCO36, in which *rif*AT6 was replaced by *rap*AT2, resulting in the production of rifamycin B and the new derivative 24-desmethyl rifamycin B. Here we describe the successful method adaptation of the PCR-targeting *Streptomyces* gene replacement approach to *Amycolatopsis mediterranei* S699 and further on the implementation of genetic modifications that enable an increased production of the derivative 24-desmethyl rifamycin B in the mutant strain DCO36. The described genetic modifications resulted in a mutant strain of DCO36 with *rifQ* deletion showing a 62% increase in 24-desmethyl rifamycin B production, while a mutant with *rifO* overexpression showed a 27% increase.

## Introduction

The Gram-positive strain *Amycolatopsis mediterranei* S699 is a member of the phylum *Actinomycetota* and capable of producing rifamycin B which belongs to a group of ansamycin antibiotic polyketides built by modular polyketide synthases type-I (PKS-I) (Kim et al., 1996; August et al., 1998; Floss and Yu, 1999; Floss et al., 2011; Kisil et al., 2021). Rifamycin provides the structural foundation for semisynthetic processes that lead to clinically important drugs, in particular rifampicin, which is used as a part of the therapy regime for curing tuberculosis, one of the world’s most infectious diseases caused by *Mycobacterium tuberculosis* (Global Tuberculosis Report 2023). Rifamycin binds to the β-subunit of the DNA-dependent RNA-Polymerase and acts bactericidal by suppressing mRNA synthesis (Wehrli et al., 1968). These derivatives have a high bioavailability in comparison to rifamycin B (Maggi et al., 2009). Rifamycin B itself has a low bioavailability and can only be used for traveler’s diarrhea (Di Stefano et al., 2011). Its complex structure allows limited structural changes which subsequently led to six clinically effective semisynthetic derivatives. Further modifications did not result in clinically usable structures (Sood et al., 2024). Semi-synthetic modifications of rifamycin B are primarily done at position -3 of the naphthoquinone ring and result in more lipophilic structures with increased bioavailability and wide-ranging indications (Cannata and Tamagnone, 1985; Maggi et al., 2009). In addition to their use against *Mycobacterium tuberculosis* these derivatives have also been used against leprosy caused by *M. leprae* as well in AIDS-related mycobacterial infection and treatment of traveler’s disease caused by *Escherichia coli* (Rees et al., 1970; Waters et al., 1978; Alvisi et al., 1987). Rifampicin was introduced into the market in 1968 and became a first-line drug within the therapeutic scheme against *M. tuberculosis* infections (Sensi, 1983; Pozniak et al., 1999). As with many antibiotics, multi-drug resistance strains of *M. tuberculosis* emerged in TB patients due to inadequate medical supervision, incorrect use, and inadequate compliance (Global Tuberculosis Report 2023). Since semi-synthetical changes of rifamycin B only led to a few clinically used rifamycin derivatives, new approaches were developed to gain additional rifamycin derivatives and overcome the problem posed by multidrug-resistant strains (MDR) of mycobacteria.

**Figure 1:**
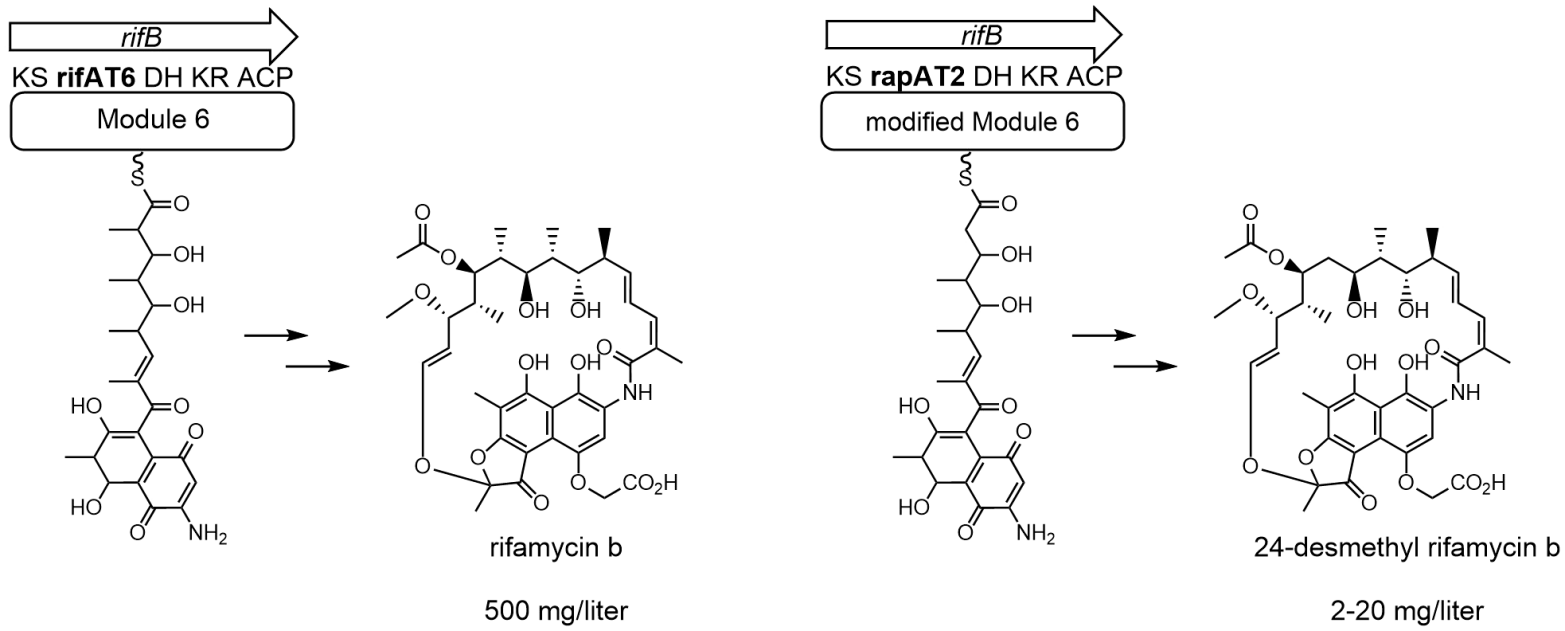
Partial rifamycin biosynthetic clusters of *Amycolatopsis mediterranei* S699 producing rifamycin B and DCO36 producing 24-desmethyl rifamycin B. The acyltransferase domain in module 6 uses methyl malonyl-CoA as an extender unit during the biosynthesis of rifamycin B for the continuous building of the polyketide backbone. In DCO36 this acyltransferase domain was exchanged by the *rap*AT2 domain. The *rap*AT2 domain uses malonyl-CoA as an extender unit and therefore the biosynthesis results in the production of 24-desmethyl rifamycin B. In comparison, *Amycolatopsis mediterranei* S699 can produce rifamycin B up to 500 mg/Liter compared to 2–20 mg/l of DCO36 (Nigam et al., 2014).

**Figure 2:**
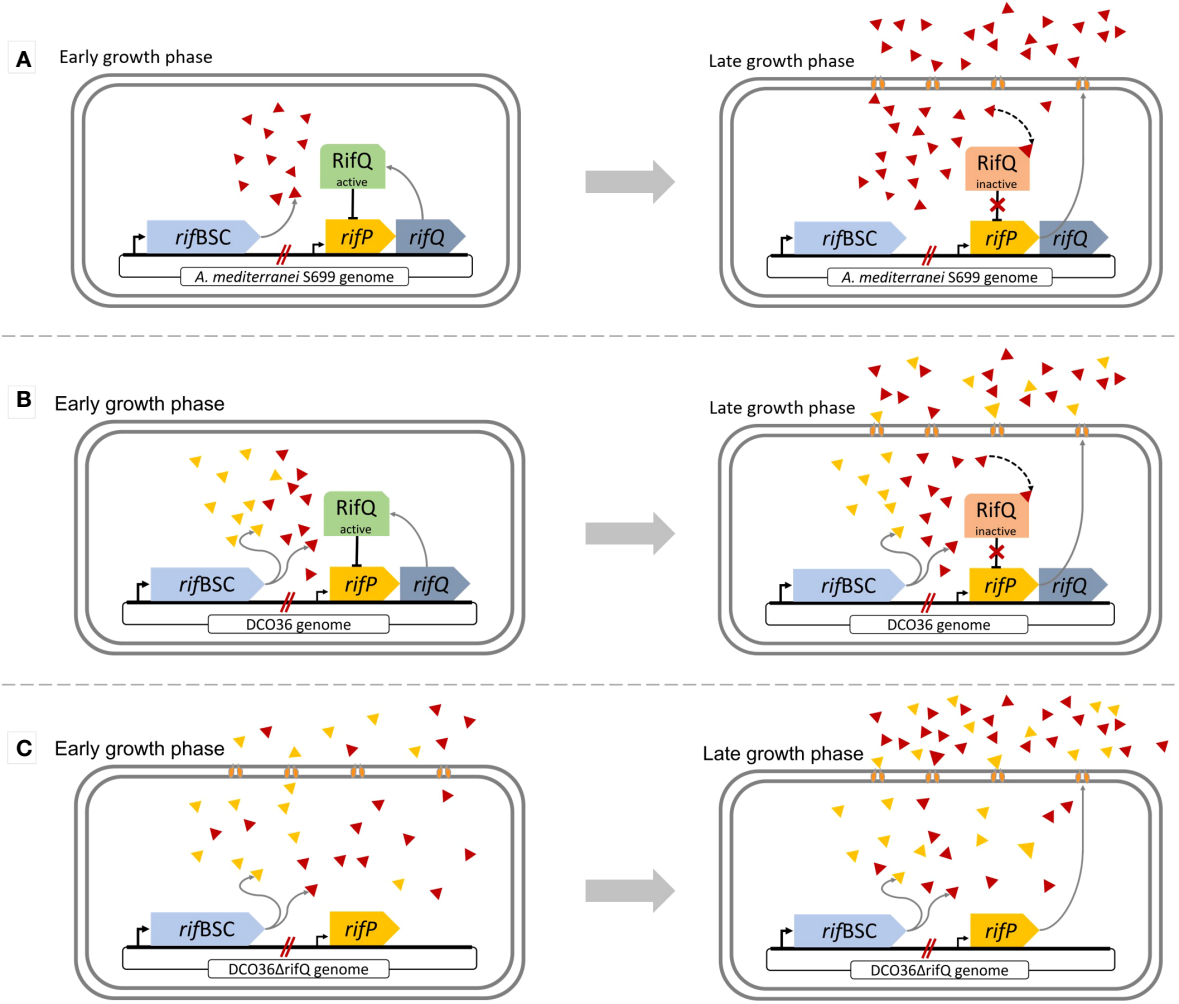
Comparison of the regulatory feedback system of rifamycin B or 24-desmethyl rifamycin B-producing strains involving the function of RifQ and RifP during different growth phases. **(A)** Wild-type regulation: At the early growth phase active RifQ (green) inhibits the expression of *rifP*. The efflux pump RifP is not produced, resulting in the accumulation of rifamycin B, which at a certain threshold of the intracellular rifamycin B concentration leads to the inactivation of RifQ (red) through its binding. As a result, the transmembrane transporter RifP is integrated into the membrane allowing the cell to transfer intracellular rifamycin into the media. **(B)** Mutant DCO36 regulation: Before the removal of *rifQ*, DCO36 shares the same regulatory feedback system as the wild-type. The only difference is the production of 24-desmethyl rifamycin B besides rifamycin B. *In silico* experiments showed that 24-desmethyl rifamycin B binds 14 times weaker to RifQ than rifamycin B. **(C)** Mutant DCO36ΔrifQ regulation: After the removal of *rifQ* resulting in DCO36ΔrifQ, the strain is already able to express *rifP* at an early growth phase. Therefore, the transmembrane transporter RifP is produced and integrated into the membrane. The transfer of rifamycin and its derivatives across the membrane right from the beginning of the growth phase, results in a higher antibiotic yield.

One approach is to directly manipulate the rifamycin polyketide biosynthetic gene cluster of the rifamycin B-producing strain *Amycolatopsis mediterranei* S699 resulting in new structural derivatives of rifamycin B, which then can also be converted into further semi-synthetical structures (Tang et al., 2012; Nigam et al., 2014). In practice, this could be done by combinatorial biosynthesis of the modular rifamycin PKS-I using homologous recombination (Lal et al., 2000). Rifamycin B is built by a modular PKS-I which consists of a loading module using 3-amino-5-hydroxy benzoic acid (AHBA) as a starter unit followed by the addition of two acetates and eight propionates by the ten extension modules (Floss and Yu, 1999; Kato et al., 2002; Floss et al., 2011). These modules are responsible for the successive polyketide chain assembly by either methyl malonyl- or malonyl-CoA. The modules differ in their composition of domains resulting in different functional groups for each of these C_2_-extender units. Responsible for the chain extension is the keto synthase (KS), acyltransferase (AT), and acyl-carrier protein (ACP) domain of each module. Depending on the composition of the module they are additionally accompanied by a dehydrogenase (DH) or keto reductase (KS) (Khosla et al., 1999). The use of combinatorial biosynthesis of *ery* PKS (erythromycin polyketide biosynthetic gene cluster) that leads to new erythromycin analogues has been amply demonstrated in *Saccharopolyspora erythreaea* (Staunton, 1998; Lü et al., 2020). In *ery* PKS the 6-deoxyerythronolide b synthase (DEBS) is equal to the rifamycin PKS-I and confirmed that DEBS domains can be swapped with each other, modified, or exchanged to domains of different PKS-I biosynthesis clusters (McDaniel et al., 1999). The rifamycin B biosynthetic gene cluster of *Amycolatopsis mediterranei* S699 was shown to be more rigid to combinatorial approaches (Floss, 2006). It could finally be manipulated resulting in the 24-desmethyl rifamycin B-producing strain DCO36 (**Figure 1**) by exchange of *rif*AT6 with *rap*AT2 (Nigam et al., 2014; Sood et al., 2024). In Lal and his coworker’s approach malonyl-CoA (*rap*AT2) was used as an extender unit instead of the original incorporation of methyl malonyl-CoA (*rif*AT6). Besides 24-desmethyl rifamycin B DCO36 still produces rifamycin B due to the assumingly broad selectivity of the new AT domain (**Figure 2B**). However, by using an antibacterial assay against rifampicin-sensitive and resistant strains of *M. tuberculosis* they were able to show that the novel 24-desmethyl rifamycin B when converted into 24-desmethyl rifampicin or 24-desmethyl rifamycin S has strong antibacterial activity. This result showed promising potential for the commercial use of these compounds. The creation of such a new derivative comes at the expense of a lower yield compared to the wild-type *Amycolatopsis mediterranei* S699. While industrial strains like *N. mediterranei* N813 can produce up to 24 g/L, these newly developed mutant strains have an antibiotic yield of 2–20 mg/L (**Figure 1**) (Lal et al., 1995; Jin et al., 2002; Nigam et al., 2014; Sood et al., 2024).

The biosynthetic gene cluster of rifamycin also contains genes encoding regulatory proteins. The deduced amino acid sequence of *rifO* is putatively involved in the regulation of the production of rifamycin by the production of the B-factor (3’-(1-butyl phosphoryl) adenosine) (**Supplementary Figure 4**) (Kawaguchi et al., 1984, 1988; Azuma et al., 1990). RifO shows similarity to 2’,3’-cyclic nucleotide 2”-phosphodiesterases. The B-factor occurs naturally and has been isolated from yeast extracts. It was shown that an external addition of the B-factor to the media can stimulate rifamycin B synthesis. This effect was already noticeable at an extremely low concentration of 10 ng/mL (Kawaguchi et al., 1984). It was mentioned by Azuma et al. that rifamycin biosynthesis was inhibited in the absence of *rifO*. Therefore, we decided to investigate whether RifO is important for rifamycin biosynthesis in *Amycolatopsis mediterranei* S699 and whether *rifO* can be used in DCO36 to increase 24-desmethyl rifamycin B production.

The protein encoded by *rifQ* is part of the feedback regulatory system of the rifamycin biosynthetic cluster (**Figure 2**) (Lei et al., 2018; Singhvi et al., 2021). The expression of *rifQ* leads to RifQ which is present as a homodimer (46.9 kDa) within the cell. RifQ is part of the TetR family and its overall structure can be broken down into two DNA-binding domains on each monomer and a regulatory core, which is responsible for the rifamycin recognition and dimerization by making hydrophobic contacts within the regulatory core (Cuthbertson and Nodwell, 2013). RifQ acts in the early growth phase as a repressor for the expression of *rifP* encoding a transmembrane transporter of the major facilitator superfamily (MFS) with 14 transmembrane domains (53 kDa) (Absalón et al., 2007; Drew et al., 2021; Singhvi et al., 2021). The hydrogen/drug antiporter (H^+^/Rifamycin) functions as an efflux pump via an electrochemical proton gradient. Rifamycin can remove repression in a later growth phase by binding to the RifQ-homodimer. A certain threshold of intracellular rifamycin concentration leads to the binding of two rifamycin molecules to the RifQ-homodimer resulting in a conformational change of RifQ. RifQ in the form of [Rifamycin-RifQ] complex loses its function as a repressor of *rifP* expression. As a consequence, *rifP* is expressed already during the early growth phase which leads subsequently to a higher overall yield of rifamycin.

Previous studies showed that the removal of *rifQ* in *Amycolatopsis mediterranei* leads to a higher antibiotic yield in comparison to the wild-type (Lei et al., 2018). Also, it was suggested that this approach may lead to a positive effect in mutant strains (Singhvi et al., 2021). More specifically, *in silico* experiments displayed that 24-desmethyl rifamycin B has a 14 times weaker binding affinity to RifQ than rifamycin B. As a consequence, expression of *rifP* is inhibited more intensely in the 24-desmethyl rifamycin B-producing mutant resulting in low production.

Here we report, besides our studies on *rifO* (see above) the generation and characterization of a *rifQ* mutant in DCO36.

**Figure 3:**
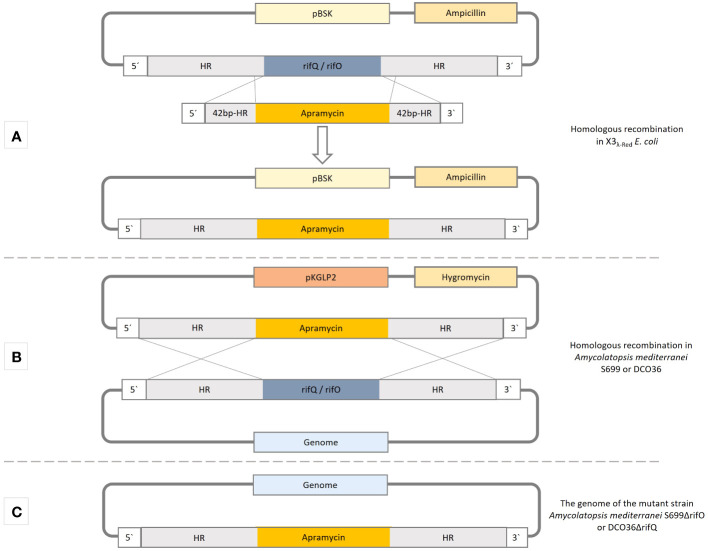
The methodical procedure of homologous recombination by the use of a modified PCR-targeted *Streptomyces* gene replacement method to replace a gene of interest in *Amycolatopsis mediterranei* strains. Carried out accordingly for *rifQ* in DCO36 and *rifO* in *Amycolatopsis mediterranei* S699. HR-homologous regions (2.5 kb). **(A)** Homologous recombination in *E coli* X3_λ-Red_ cells to exchange the gene of interest (*rifQ*/*rifO*) and replace it with apramycin resistance cassette of pLERE to obtain pBSK-apraR-HR_(_ *_rifQ/ rifO_*_)_. **(B)** The construct apraR-HR_(_ *_rifQ/ rifO_*_)_ is integrated into the non-replicative suicidal *Streptomyces* vector pKLGP2 and transformed into ET_12567/pUZ8002_ for intergeneric conjugation with *Amycolatopsis mediterranei* S699 or DCO36. **(C)** Resulting genome after the replacement of the gene of interest (*rifQ*/*rifO*) with the apramycin resistance cassette (ApraR).

This study demonstrates the adaptation of the modified PCR-targeted *Streptomyces* gene replacement method to remove or exchange genes in *Amycolatopsis mediterranei* S699 and its mutant strains like the 24-desmethyl rifamycin B-producing strain DCO36 (**Figure 3**) (Gust et al., 2003). For *Streptomyces* species a wide range of suitable cloning vectors and transformation methods are well established (Kieser et al., 2000). Nonetheless, the adaptation of these methods and plasmids to *Amycolatopsis mediterranei* S699 is an extremely difficult process that does not always necessarily lead to the desired success (Lal et al., 1991). The development of suitable plasmids is also a very time-consuming procedure (Tuteja et al., 2000; Dhingra et al., 2003). The combinative use of the suicidal vector pKGLP2 and the replicative and consecutive PermE promoter-containing plasmids pUWL-H (respectively pUWL-HA), shown in this study, enables a reliably quick way to switch off, exchange, or specifically overproduce genes (Petzke, 2010; Myronovskyi et al., 2011). Furthermore, the results indicate the potential that the successful implementation of the modified PCR-targeted *Streptomyces* gene replacement method and use of the mentioned plasmids will have on previous time-consuming and problematic approaches to manipulate rifamycin polyketide synthase gene cluster or the modification of regulatory elements of *Amycolatopsis mediterranei* strains. The adaptation of the method and plasmids led subsequently to the presented results in this study.

This study investigated whether, for the 24-desmethyl rifamycin B derivative-producing strain DCO36, the removal of *rifQ* leads to increased production of rifamycin B derivative. After the removal of *rifQ*, the respective strain can produce and integrate the transmembrane transporter RifP into the membrane already at the early growth phase. This can lead to a higher yield of 24-desmethyl rifamycin B as the intracellular concentration does not need to reach the threshold concentration to inactivate RifQ.

This study also investigated the role of RifO and its involvement in the regulation of rifamycin B production by its production of the B-Factor. With the removal of *rifO* in *Amycolatopsis mediterranei* S699, the production of rifamycin B should be decreased when RifO plays a crucial role in the production of rifamycin B and its derivatives.

This study investigated the potential of an internal increase of B-Factor as it was shown by Kawaguchi et al., that the external addition of B-Factor to the media stimulates the rifamycin B synthesis of cells. This led to the approach to enable cells to constitutively overexpress *rifO* followed by an intrinsic rise of B-factor, which itself can increase rifamycin production. This potential positive effect of constitutive overexpression of *rifO* on the rifamycin or its derivatives production was investigated for DCO36.

## Materials and methods

### Chemicals

All chemicals were obtained by Carl Roth GmbH + Co. KG (Karlsruhe, DE).

### Bacterial strains, plasmids, and culture media

*Actinomyces* strain *Amycolatopsis mediterranei* S699 (Tang et al., 2012) was used for the removal of *rifO* and mutant strain DCO36 producing 24-desmethyl rifamycin B was used for the removal of *rifQ* and the overexpression of *rifO*. In this work, different *Escherichia coli* strains were used for the following tasks. *E. coli* XL-1 Blue strain for genetic work, *E. coli* X3_λ-Red_ (*E. coli* BW25113) for homologous recombination, and *E. coli* ET_12567/pUZ8002_ for intergeneric conjugation with *Amycolatopsis mediterranei* S699 and DCO36. To remove *rifQ* plasmids pBSK-rifQ-HR, pBSK-apraR-HR_rifQ,_ and pKGLP2-apraR-HR_rifQ_ were created. To remove *rifO* plasmids pBSK-rifO-HR, pBSK-apraR-HR_(rifO),_ and pKGLP2-apraR-HR_rifQ_ were created (**Figure 3**; **Supplementary Table 1**). For the overexpression of *rifO*, pUWL-HA-rifO was created (**Supplementary Table 1**). All plasmids used for subcloning are based on the pBluescript II KS(+) vector (pBSK). The vector pLERE contains the apramycin resistance cassette (apraR) which was used for the replacement of genes (Herrmann et al., 2012). For the removal of *rifQ* or *rifO* by homologous recombination in DCO36 or *Amycolatopsis mediterranei* S699 the suicidal non-replicative vector pKGLP2 was used in this work. For the overexpression of *rifO*, the replicative *Streptomyces* vector pUWL-HA containing a consecutive PermE-promoter and apramycin resistance cassette was used. Restriction enzymes were used for the individual cloning steps in order to generate the described plasmids and, if important, are mentioned in the appropriate places below. Bacterial strains derived from *E. coli* were cultivated in LB-media and incubated in a rotary shaker overnight (37°C, 180 rpm). Bacterial strains derived from *Amycolatopsis mediterranei* S699 were cultivated on YMG agar plates or in YMG media and incubated in a rotary shaker (28°C, 180 rpm).

### Construction of plasmids and genetic manipulation

For the removal of *rifQ* and *rifO*, a modified protocol of the PCR-targeted *Streptomyces* gene replacement approach was applied (**Figure 3**) (Gust et al., 2003).

### Generation of DCO36ΔrifQ

The target gene *rifQ* was amplified from the genomic DNA of *Amycolatopsis mediterranei* S699 together with an additional 2.2–2.3 kbp homologous region (HR) up and downstream of the concerning gene (**Figure 3**; **Supplementary Table 1**). The product *rifQ*-HR was ligated into the pBluescript II KS(+) vector to obtain pBSK-rifQ-HR, which was transformed into *E. coli* X3_λ-Red_ -cells containing a λ-Red system (Gust et al., 2003). A short PCR product (apraR-sHR) with an apramycin resistance cassette, flanked by two 42 base pairs of the homologous regions (sHR) up and downstream of *rifQ* was amplified from pLERE (**Supplementary Table 1**) (Herrmann et al., 2012). The PCR product was electroporated into *E. coli* X3_λ-Red_/pBSK-rifQ-HR cells (**Supplementary Table 1**). The homologous recombination between the pBSK-rifQ-HR and apraR-sHR requires the cells λ-Red System, which was induced with 1% arabinose (V/V). The resulting plasmid pBSK-apraR-HR_rifQ_ was isolated, the apraR-HR_rifQ_ region amplified and ligated into pKGLP2, resulting in pKGLP2-apraR-HR_rifQ_ which was then transformed into *E. coli* ET_12567/pUZ8002_ cells (**Supplementary Table 1**). As pKGLP2 is a suicide vector in *Streptomyces*, but not in *E. coli*, it can be replicated by them and then transferred to the mutant strain DCO36 of *Amycolatopsis mediterranei* by intergeneric conjugation. The sequencing of the isolated genomic DNA of DCO36 revealed a successful replacement of *rifQ* by the apramycin resistance cassette. The resulting strain was named DCO36ΔrifQ (DCO36::apraRΔrifQ) (**Figure 3**).

### Generation of *Amycolatopsis mediterranei* S699ΔrifO

The target gene *rifO* was amplified from the genomic DNA of *Amycolatopsis mediterranei* S699 together with an additional 2.2–2.3 kbp homologous region (HR) up and downstream of the concerning gene (**Figure 3**; **Supplementary Table 1**). The product *rifO*-HR was ligated into the pBluescript II KS(+) vector to obtain pBSK-rifO-HR, which was transformed into *E. coli* X3_λ-Red_ -cells containing a λ-Red system (Gust et al., 2003). A short PCR product (apraR-sHR) with an apramycin resistance cassette, flanked by two 42 base pairs of the homologous regions (sHR) up and downstream of *rifO* was amplified from pLERE (**Supplementary Table 1**) (Herrmann et al., 2012). The PCR product was electroporated into *E. coli* X3_λ-Red_/pBSK-rifO-HR. The homologous recombination between the pBSK-rifO-HR and apraR-sHR requires the cells λ-Red System, which was induced with 1% arabinose (V/V). The resulting plasmid pBSK-apraR-HR_rifO_ was isolated, the apraR-HR_rifO_ region amplified and ligated into pKGLP2, resulting in pKGLP2-apraR-HR_rifO_ which was then transformed into *E. coli* ET_12567/pUZ8002_ cells (**Supplementary Table 1**). As pKGLP2 is a suicide vector in *Streptomyces*, but not in *E. coli*, it can be replicated by them and then transferred to the mutant strain DCO36 of *Amycolatopsis mediterranei* by intergeneric conjugation. The sequencing of the isolated genomic DNA of DCO36 revealed a successful replacement of *rifO* by the apramycin resistance cassette. The resulting strain was named *Amycolatopsis mediterranei*ΔrifO (*Amycolatopsis mediterranei* S699::apraRΔrifO) (**Figure 3**).

### Generation of DCO36-rifO (DCO36/pUWL-HA-rifO)

The target gene *rifO* was amplified from the genomic DNA of *Amycolatopsis mediterranei* S699 by using forward/reverse primers containing *HindIII*/*XbaI* cleavage sites to ligate HindIII-rifO-XbaI into pUWL-H after digestion (**Supplementary Table 1**). The vector pUWL-H contains a strong constitutive erythromycin promoter (PermE), which enables a high expression of *rifO* and a hygromycin resistance gene for selection. Hygromycin was not used for selection due to insufficient selection. For selection, apramycin was used. The apramycin resistance cassette (apraR) was amplified from pLERE and integrated into pUWL-H-rifO using *NdeI* cleavage sites to obtain the final plasmid pUWL-HA-rifO. This plasmid was transformed into ET_12567/pUZ8002_ for intergeneric conjugation with DCO36 to receive the resulting strain named DCO36-rifO (DCO36/pUWL-HA-rifO).

### Growing conditions for *Actinomyces*

For the comparison of the 24-desmethyl rifamycin B or rifamycin b, production strains were grown as follows. The strains *Amycolatopsis mediterranei* S699, DCO36, DCO36ΔrifQ, DCO36/pUWL-HA-rifO, and *Amycolatopsis mediterranei* S699ΔrifO were cultivated to make conclusions regarding their growth behavior and antibiotic yield in the form of rifamycin B and 24-desmethyl rifamycin. YMG-media (150 mL) was inoculated with a well-grown preculture (2% V/V) of the mentioned strains. Cultures were grown for 4 days in a rotary shaker (28°C, 180 rpm).

### Extraction and analysis of rifamycin B and 24-desmethyl rifamycin B

Grown cultures were harvested by centrifugation and pellets were discarded. The supernatant was adjusted with 1 M HCl to a pH of 3.5 and extracted with ethyl acetate (200% V/V). The lipophilic phase was evaporated until dry. Extracts were dissolved in 1 mL methanol and filtered through a 0.22 μm particle filter before analysis with high-pressure liquid chromatography-mass spectrometry (HPLC-MS). 100 μL of the extract was transferred to an HPLC vial for measurement. The injection volume was 1 μL. For analysis Waters XBridge^®^ C18 main-column (4.6 x 100 mm, 3.5 µm) and Waters XBridge^®^ C18 pre-column (4.6 x 20 mm, 5 µm) were used. The masses were recorded using a mass spectrometer with electrospray ionization (ESI) and a quadrupole mass detector in negative mode (**Supplementary Figure 3**). The *rifamycin_neg* method was used for the extracts (**Supplementary Figure 2**).

## Results

### Application of the PCR-targeted *Streptomyces* gene replacement approach to *Amycolatopsis mediterranei* S699 and DCO36

In this study, we show that PCR-targeted *Streptomyces* gene replacement can be applied to efficiently remove genes in *Amycolatopsis mediterranei* S699 and its mutant strains DCO36. For the removal of *rifQ* and *rifO* described in the paper´s method section, a modified protocol of the PCR-targeted *Streptomyces* gene replacement approach was applied (**Figure 3**) (Gust et al., 2003).

### Generation of a *rifO*-mutant of *Amycolatopsis mediterranei* S699

**Figure 4:**
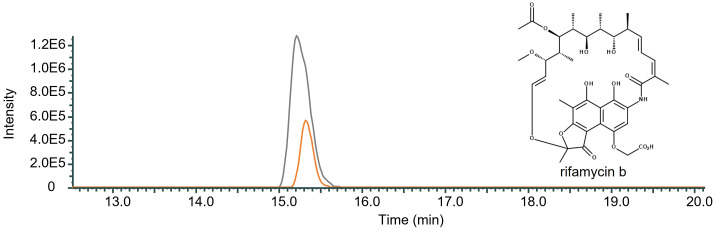
Qualitative comparison of the impact of the removal of *rifO* in rifamycin B producing wild-type strain *Amycolatopsis mediterranei* S699. The single mass-ion chromatogram shows the characteristic peak of rifamycin B m/z 754 [M-H] ^─^ at its retention time (Rt 15.30 min) resulting from HPLC-electrospray ionization mass spectrometry analysis of *Amycolatopsis mediterranei* S699 (AUC = 6.178E6; grey) and *Amycolatopsis mediterranei* S699ΔrifQ (AUC = 2.102E7; orange) extracts (Spectrum, **Supplementary Figure 3**). The comparison shows the decreased amount of rifamycin B after the removal of *rifO* in the wild-type strain *Amycolatopsis mediterranei* S699 by 70.61%.

In order to study the influence of RifO on rifamycin production in *Amycolatopsis mediterranei* S699 a *rifO* mutant was generated. The mutant showed a decrease in rifamycin B production by 70.61% compared to the wild-type *Amycolatopsis mediterranei* S699 indicating that RifO is needed for efficient production (**Figure 4**).

### Generation of a *rifO*-overexpressing mutant of DCO36

**Figure 5:**
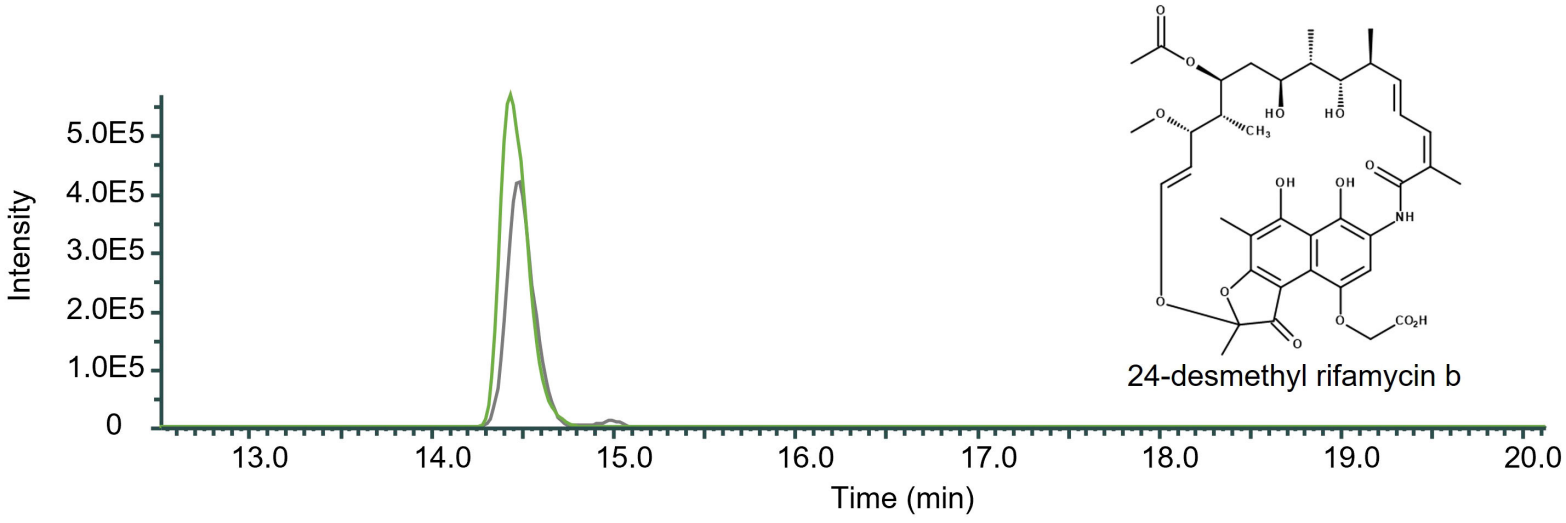
Qualitative comparison of the impact of *rifO* overexpression in 24-desmethyl rifamycin B-producing mutant strain DCO36. The single mass-ion chromatogram shows the increased amount of 24-desmethyl rifamycin B after the overexpression of *rifO* in a rifamycin B derivative-producing mutant strain resulting from HPLC-electrospray ionization mass spectrometry analysis of DCO36 (AUC = 4.485E6; grey) and DCO36-rifO (AUC = 6.164E6; green) extracts. The chromatographs show the characteristic peak of the derivative 24-desmethyl rifamycin B m/z 740 [M-H] ^─^ at its retention time (Rt 14.50 min) (Spectrum, **Supplementary Figure 3**). The comparison shows the increased amount of 24-desmethyl rifamycin B after the overexpression in a rifamycin B derivative-producing mutant strain by 27.24%.

As RifO is a positive regulator for rifamycin production we decided to overexpress it in the mutant DCO36 resulting in DCO36-rifO. As shown by HPLC-MS the strain showed an increase in 24-desmethyl rifamycin B formation by 27.24% compared to the 2–4 mg/mL 24-desmethyl rifamycin B-producing DCO36 (**Figure 5**). It can be stated that the internal and previously shown external increase of the B-factor has a positive impact on the rifamycin B or exemplary 24-desmethyl rifamycin B production.

### Generation of a *rifQ*-mutant of DCO36

**Figure 6:**
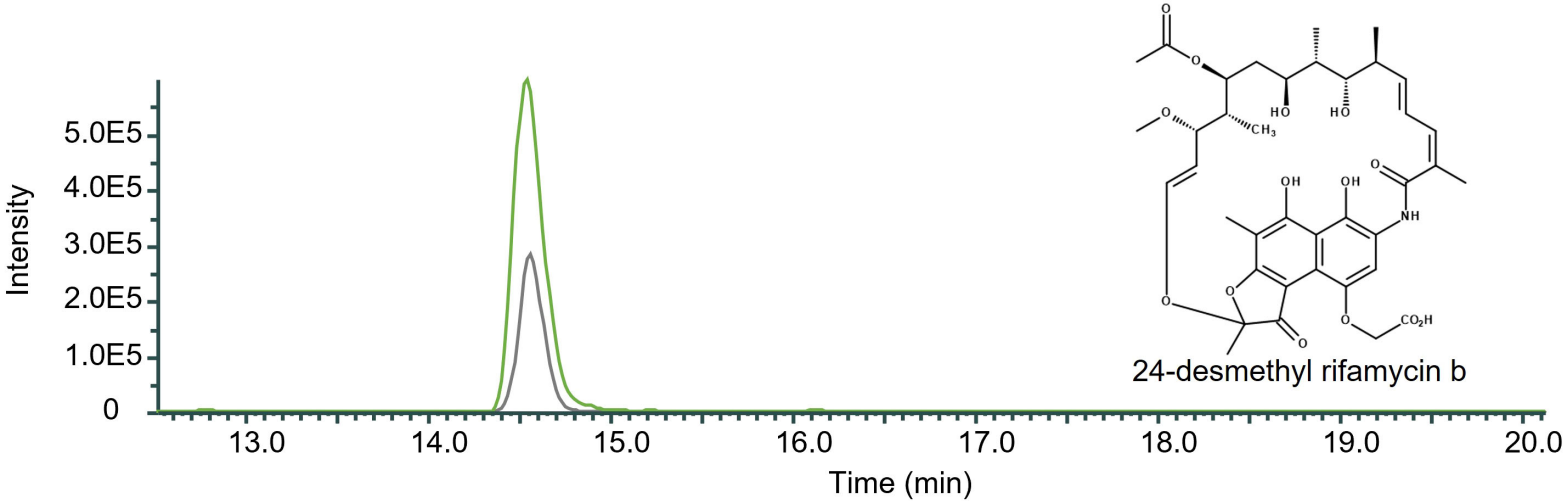
Qualitative comparison of the impact of the removal of *rifQ* in 24-desmethyl rifamycin B-producing mutant strain DCO36. The single mass-ion chromatogram shows the characteristic peak of the derivative 24-desmethyl rifamycin B m/z 740 [M-H] ^─^ at its retention time (Rt 14.50 min) (**Supplementary Figure 3**) resulting from HPLC-electrospray ionization mass spectrometry analysis of DCO36 (AUC = 2.722E6; grey) and DCO36ΔrifQ (AUC = 7.083E6; green) extracts (Spectrum, **Supplementary Figure 3**). The comparison shows the increased amount of 24-desmethyl rifamycin B after the removal of *rifQ* in a rifamycin B derivative-producing mutant strain by 61.57%.

The removal of *rifQ* resulted in the mutant strain DCO36ΔrifQ, which showed an increase in 24-desmethyl rifamycin B production by 61.57% compared to the 2–4 mg/mL 24-desmethyl rifamycin B producing DCO36 (**Figure 6**).

## Discussion

This study illustrates the effective adaptation of a modified PCR-targeted *Streptomyces* gene replacement technique for gene manipulation in *Amycolatopsis mediterranei* S699 and its mutant strains, including DCO36, known for producing 24-desmethyl rifamycin B. While *Streptomyces* species benefit from a diverse array of established cloning vectors and transformation methods, applying these techniques to *Amycolatopsis mediterranei* S699 proves notably challenging, with success not always guaranteed (Lal et al., 1991; Kieser et al., 2000). The development of suitable plasmids for this purpose is a time-intensive process (Tuteja et al., 2000; Dhingra et al., 2003). However, by utilizing the suicidal vector pKGLP2 in conjunction with replicative and consecutive PermE promoter-containing plasmids like pUWL-H (and pUWL-HA), this study offers a novelty reliable and efficient method for gene manipulation, facilitating the quick switching off, exchanging, or targeted overproduction of genes in *Amycolatopsis mediterranei* strains. These findings hint at the potential impact of this adapted method and plasmid use in streamlining earlier laborious approaches to modifying the rifamycin polyketide synthase gene cluster or altering regulatory elements in *Amycolatopsis mediterranei* strains. The successful adaptation of this method and plasmids paved the way for the results presented in this study.

As the removal of *rifQ* and the overexpression of *rifO* lead to an increase in *Amycolatopsis mediterranei* S699 mutant strains of rifamycin B derivatives, these genetic modifications can, in general, be seen as new useful tools to increase production like it was previously only shown for the wild type by the removal of *rifQ* (Lei et al., 2018). Based on the impact of these modifications a combination of the removal of *rifQ* as well as a simultaneous overproduction of *rifO* could further increase 24-desmethyl rifamycin B production. The approaches might also be usable in upcoming rifamycin B derivative-producing mutant strains. A developing understanding and further studies of factors and genes involved in the induction or increase of the biosynthetic cluster of rifamycin and its derivatives can lead to further genetic modifications to increase antibiotic yield.

To quantify the antibiotic production rate of rifamycin B derivative-producing strains, these mutants should be grown in the fermenter to gain a better quantitative understanding. Since the growth of these strains depends on multiple factors like dissolved oxygen concentration, glucose concentration, and pH during the growth or building of foam which secondly influences the production of rifamycin B and its derivatives, the growth in the fermenter is an interesting aspect for these optimized mutant strains. This allows further quantitative statements to be made, such as the antibiotic production performance in g/L, which can further expand the results presented in this work of the genetic changes carried out in mutant strains.

The removal of *rifQ* and the overexpression of *rifO* resulting in higher antibiotic yields in mutant strains can potentially close the gap to commercial use of their rifamycin B derivatives like it is aimed for 24-desmethyl rifamycin B of DCO36 (Sood et al., 2024).

## Data availability statement

The original contributions presented in the study are included in the article/**Supplementary Material**, further inquiries can be directed to the corresponding author/s.

## Author contributions

MM: Conceptualization, Investigation, Methodology, Writing – original draft. EB: Methodology, Writing – review & editing. IS: Investigation, Methodology, Writing – original draft. US: Writing – review & editing. RL: Conceptualization, Writing – review & editing. AB: Conceptualization, Funding acquisition, Methodology, Project administration, Resources, Supervision, Writing – original draft, Writing – review & editing.
