# Supplementary material for: Genomic modifications for enhanced antibiotic production in rifamycin derivative-producing Amycolatopsis mediterranei S699 strains: focusing on rifQ and rifO genes
Source: Front Antibiot. 2024 Jun 24;3:1399139. doi: 10.3389/frabi.2024.1399139 (PMC11732027; doi:10.3389/frabi.2024.1399139)
Supplement: Supplementary file 1 [file DataSheet_1.docx]

Supplementary Material

**Supplementary Table 1. Primer used in this work.**

| **Name** | **Sequence**  **5´-3´** | **Template** |
| --- | --- | --- |
|  | **Removal of *rifQ*** |  |
| rifQ-HR_for_1 | CCCATCGCCTACCAGTTCCTGAACG | Genomic DNA  *Amycolatopsis* *mediterranei* S699 |
| rifQ-HR_rev_2 | CGACCACCTACCTGATCGGCAACTC |  |
| Apra_rifQ_for | CCTGCCCCCTTTACCGTTGACCTCCGGTGAGAAGGGAGGGCCACGATGGATATCTCTAGATACCG | pLERE |
| Apra_rifQ_rev | TCACCCGCCGGCGACCATGCCGGTGAACAGCCAGTTCAGCCCTCAAACAAAAGCTGGAGCTC |  |
| DCO-apraR/rifQ-for | CTCGTCATGGGTGGCGGTATC | Genomic DNA DCO36ΔrifQ |
| DCO-apraR/rifQ-rev | GTCGACTACGGCAAGACGTCGAAC |  |
|  | **Removal of *rifO*** |  |
| rifO-HR_for | GGATCTCCGTCTTCCTCGAGAAGC | Genomic DNA  *Amycolatopsis* *mediterranei* S699 |
| rifO-HR_rev | CGGGATCGAGGTCATGATGG |  |
| Apra_rifO_for_1 | ATGGGCACTCTCGTTTCCTTCCACGCACACCCCAACGACGACGATATCTCTAGATACCG | pLERE |
| Apra_rifO_rev_2 | TTACGCCATCAGGTCGGTTTCGGTGATGCCCGGCCCCTGACCAACAAAAGCTGGAGCTC |  |
| DCO-apraR/rifO-for | CTGCGAGAACCTGCTGTACATCG | Genomic DNA DCO36ΔrifO |
| DCO-apraR/rifO-rev | GCTGATGCCGTACGTGAC |  |
|  | **Overexpression of *rifO*** |  |
| rifO_HindIII_for | GTACCaagcttATGGGCACTCTCGTTTCC | Genomic DNA  *Amycolatopsis* *mediterranei* S699 |
| rifO_XbaI_rev | GAtctagaGTTACGCCATCAGGTCG |  |

**Supplementary Table 2. Analytical HPLC-MS method *rifamycin_neg*.** Flow rate 0.5 mL/min, column oven 30°C. Mobile phase A: Acetonitrile + 0.5% acetic acid (V/V); Mobile phase B: ddH_2_O + 0.5% acetic acid (V/V).

| Time [min] | Mobile phase A [%] | Mobile phase B [%] |
| --- | --- | --- |
| 0 – 5 | 35 | 65 |
| 5 – 25 | 80 | 20 |
| 25 – 28 | 95 | 5 |
| 28 – 33 | 35 | 65 |


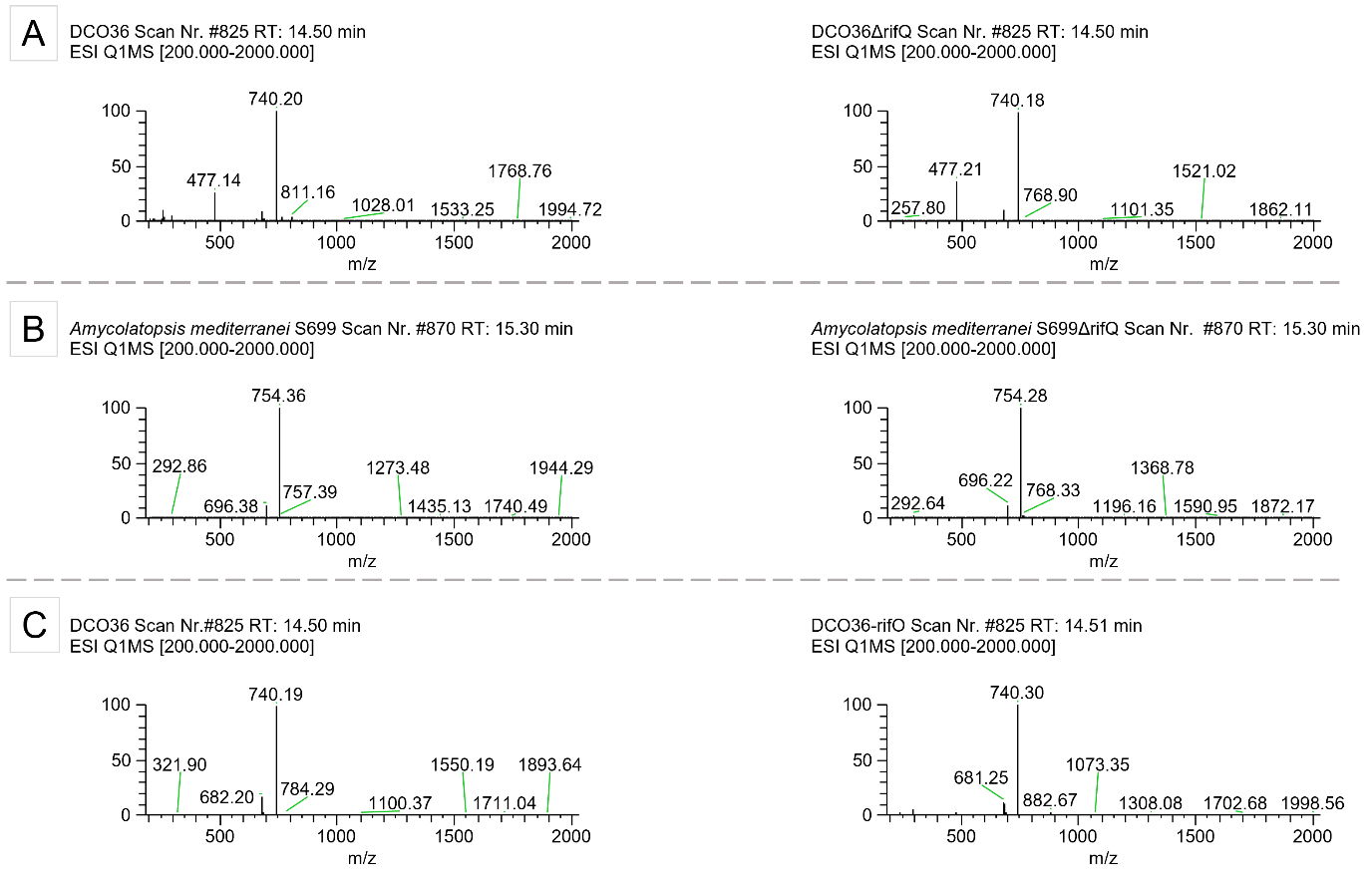


**Supplementary Figure 3. Electrospray ionization mass spectrum from HPLC-MS.** To receive the spectra of A, B, and C, high-pressure liquid chromatography-mass spectrometry (HPLC-MS) with negative electro-spray ionization was used. (**A)** The spectrum of DCO36 (left) and DCO36ΔrifQ (right) extracts show the characteristic signal of derivative 24‑desmethyl rifamycin b (m/z 740 [M‑H]^─^ at rt 14.5 min). (**B)** The spectrum of *Amycolatopsis* *mediterranei* S699 (left) and *Amycolatopsis mediterranei*699ΔrifO (right) extracts show the characteristic signal of rifamycin b (m/z 754 [M‑H]^─^ at rt 15.30 min). (**C)**The spectrum of DCO36 (left) and DCO36-rifO (right) extracts show the characteristic signal of derivative 24‑desmethyl rifamycin b (m/z 740 [M‑H]^─^ at rt 14.5 min).


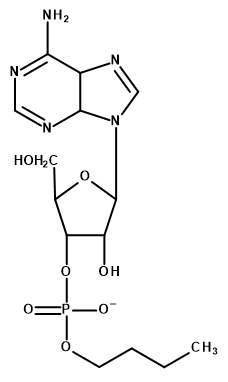


**Supplementary Figure 4. Structure of 3´-(1-butylphosphoryl) adenosin (B-factor). B‑factor is produced under the influence of RifO.** The external addition of 3´-(1-butyl phosphoryl) adenosine to media can increase rifamycin production at an extremely low concentration (10 ng/mL).
